# A Comprehensive Review on the Intricate Interplay between COVID-19 Immunization and the New Onset of Pemphigus Foliaceus

**DOI:** 10.3390/vaccines12080857

**Published:** 2024-07-30

**Authors:** Beatrice Bălăceanu-Gurău, Adrian Dumitrascu, Călin Giurcăneanu, Raluca Tatar, Cristian-Dorin Gurău, Olguța Anca Orzan

**Affiliations:** 1 Department of Oncologic Dermatology, “Elias” Emergency University Hospital, “Carol Davila” University of Medicine and Pharmacy, 020021 Bucharest, Romania; calin.giurcaneanu@gmail.com (C.G.); olguta.orzan@umfcd.ro (O.A.O.); 2 Clinic of Dermatology, “Elias” Emergency University Hospital, 011461 Bucharest, Romania; 3 Division of Hospital Internal Medicine, Department of Medicine, Mayo Clinic Florida, Jacksonville, FL 32224, USA; dumitrascu.adrian@mayo.edu; 4 Department of Plastic Reconstructive Surgery and Burns, “Grigore Alexandrescu” Clinical Emergency Hospital for Children, “Carol Davila” University of Medicine and Pharmacy, 020021 Bucharest, Romania; 5 Department of Plastic Reconstructive Surgery and Burns, “Grigore Alexandrescu” Clinical Emergency Hospital for Children, 010621 Bucharest, Romania; 6 Orthopedics and Traumatology Clinic, Clinical Emergency Hospital, 014451 Bucharest, Romania; gurau_dorin@yahoo.com

**Keywords:** coronavirus disease, COVID-19 vaccination, mRNA vaccines, autoimmunity, autoimmune bullous diseases, pemphigus foliaceus

## Abstract

Autoimmune bullous diseases (AIBDs) are characterized by the formation of vesicles, bullous lesions, and mucosal erosions. The autoantibodies target the cellular anchoring structures from the surface of epidermal keratinocyte named desmosomes, leading to a loss of cellular cohesion named acantholysis. AIBDs are classified into intraepidermal or subepidermal types based on clinical features, histological characteristics, and immunofluorescence patterns. Pemphigus foliaceus (PF) is an acquired, rare, autoimmune skin condition associated with autoantibodies that specifically target desmoglein-1, leading to a clinical presentation characterized by delicate cutaneous blisters, typically sparing the mucous membranes. Several factors, including genetic predisposition, environmental triggers, malignancies, medication use, and vaccination (for influenza, hepatitis B, rabies, tetanus, and more recently, severe acute respiratory syndrome Coronavirus 2 known as SARS-CoV-2), can potentially trigger the onset of pemphigus. With the advent of vaccines playing a pivotal role in combatting the 2019 coronavirus disease (COVID-19), extensive research has been conducted globally to ascertain their efficacy and potential cutaneous adverse effects. While reports of AIBDs post-COVID-19 vaccination exist in the medical literature, instances of PF following vaccination have been less commonly reported worldwide. The disease’s pathophysiology is likely attributed to the resemblance between the ribonucleic acid (RNA) antigen present in these vaccines and cellular nuclear matter. The protein produced by the BNT-162b2 messenger ribonucleic acid (mRNA) vaccine includes immunogenic epitopes that could potentially trigger autoimmune phenomena in predisposed individuals through several mechanisms, including molecular mimicry, the activation of pattern recognition receptors, the polyclonal stimulation of B cells, type I interferon production, and autoinflammation. In this review, we present a comprehensive examination of the existing literature regarding the relationship between COVID-19 and PF, delving into their intricate interactions. This exploration improves the understanding of both pemphigus and mRNA vaccine mechanisms, highlighting the importance of close monitoring for PF post-immunization.

## 1. Introduction

Pemphigus encompasses a rare and severe group of IgG-mediated autoimmune bullous diseases (AIBDs), with its name being derived from the Greek term “pemphix,” signifying blister or bubble formation [1,2,3]. The severity of the disease arises from its progressive nature, leading to extensive skin surface involvement and resulting in heightened catabolism, loss of body fluids and proteins, and susceptibility to secondary bacterial and viral infections, potentially culminating with sepsis (usually secondary to *Staphylococcus aureus*) and cardiac failure [1,3].

Pemphigus can be classified into two principal types: vulgaris and foliaceus [1,2,3,4,5]. Patients afflicted with pemphigus vulgaris (PV) typically present with mucosal erosions alongside cutaneous blisters and erosions [1,2,3,4,5]. These blisters arise within the deeper layers of the epidermis, situated just above the basal cell layer [1,2,3,4,5]. 

**Figure 1:**
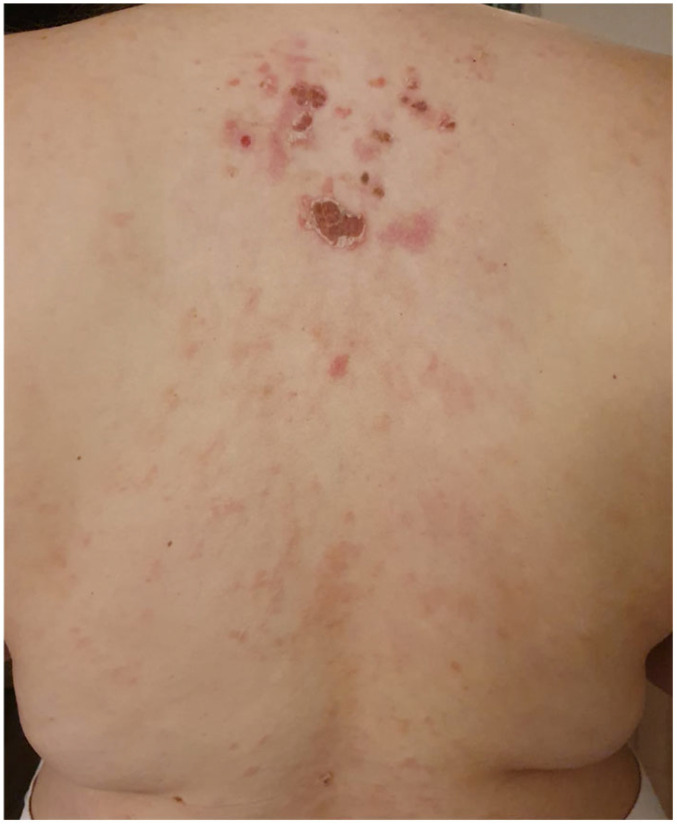
Crusted erosions localized on an erythematous base on the upper trunk.

**Figure 2:**
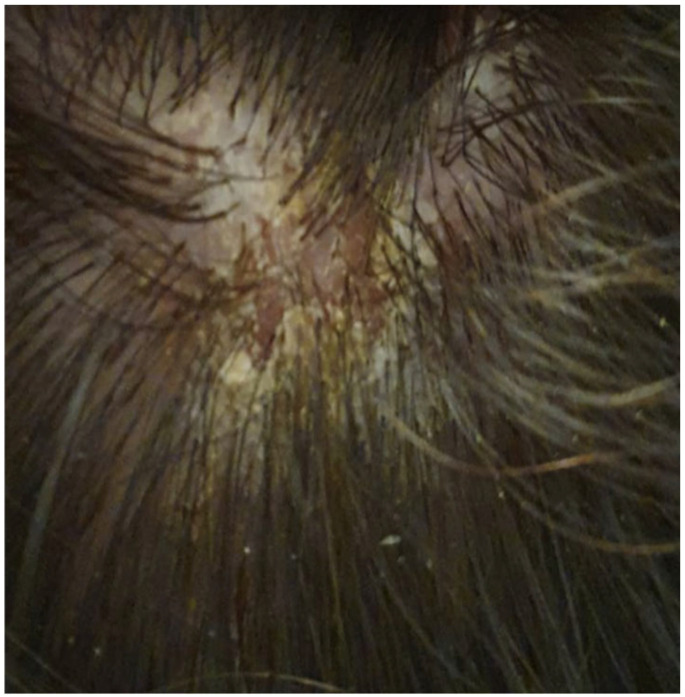
Crusted erosions placed on an erythematous base on the scalp.

**Figure 3:**
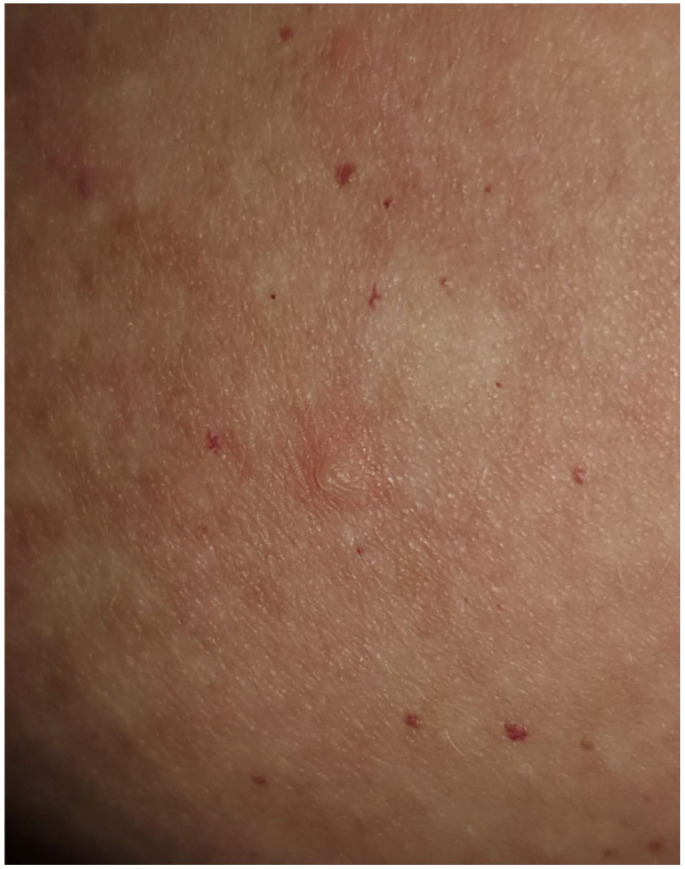
Transient small flaccid blister located on the left breast.

Pemphigus foliaceus (PF) is a well-defined cutaneous eruption characterized by transient, flaccid, superficial bullae formed within the granular layer of the epidermis, accompanied by pain and pastry-like exfoliation evolving into fragile, crusty erosions on an erythematous base, primarily observed in the seborrheic areas of the skin, including the scalp, face, and trunk, with no mucosal involvement [1,2,3,4,5] (Figure 1, Figure 2 and Figure 3).

Pathophysiologically, the formation of intraepithelial blisters in PF is caused by IgG autoantibodies binding to desmosomal adhesion proteins (particularly desmoglein-1) present on epidermal keratinocytes [1,2,3,4,5]. The attachment of autoantibodies to desmoglein-1 disrupts desmosome function and results in the loss of cell–cell adhesion within the epidermis, triggering acantholysis, a process characterized by cell detachment and the formation of intraepithelial blisters or pustules within the superficial layers of the skin [1,2,3,4,5]. The pathological role of anti-desmoglein-1 IgG antibodies is well-established, as evidenced by the development of pemphigus-like symptoms when patients’ sera or purified IgG are injected into neonatal mice [1]. In most cases, disease activity is closely linked to serum levels of desmoglein-reactive autoantibodies [1]. The presence of autoantibodies triggers a localized immune response, which contributes to the inflammatory process and exacerbates skin damage [1,2,3,4,5,6].

**Figure 4:**
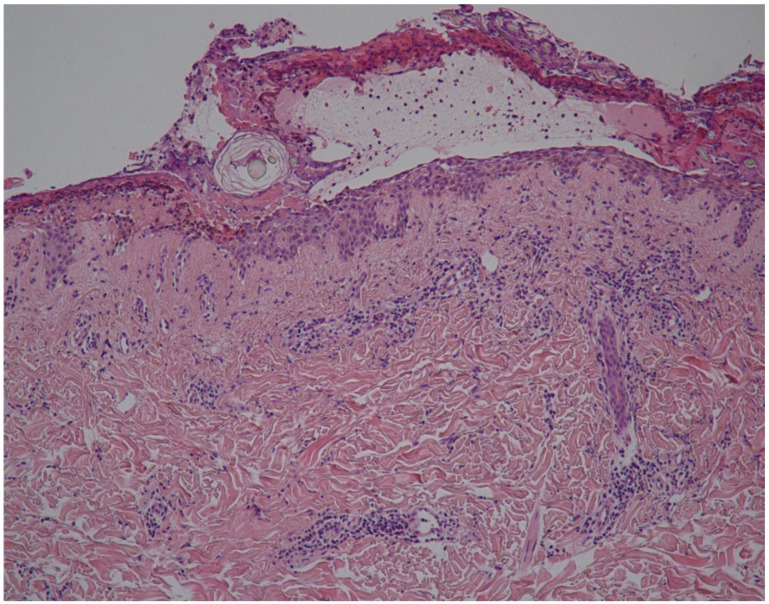
Histopathological examination reveals detachment of the upper layers of the epidermis from the lower ones, forming a suprabasal bulla with infiltrating neutrophils and focal detachment of rounded keratinocytes from the upper layers (acantholysis at the granular layer).

Microscopic examination of skin biopsies from affected areas reveals the detachment of the upper layers of the epidermis from the lower ones, forming a suprabasal bulla with infiltrating neutrophils and focal detachment of rounded keratinocytes from the upper layers (acantholysis at the granular layer). The peri-vascular and interstitial inflammatory infiltrate comprises leukocytes and neutrophils (Figure 4) [1,2,3,4,5].

Immunofluorescence microscopy shows epidermal reticular deposition of IgG and complement component 3 (C3), with the absence of IgM, IgA, and fibrinogen deposits. Complement components bind to desmoglein-1 within the intercellular spaces of the epidermis [1,2,3,4,5].

Owing to its rarity, the evidence regarding pemphigus treatment primarily stems from a limited number of prospective controlled clinical trials, often constrained by small sample sizes and a lack of statistically significant differences [1]. Various treatment options have been investigated, including different doses of prednisolone, intravenous corticosteroid pulses, and additional immunomodulant therapies with azathioprine, methotrexate, mycophenolate mofetil, cyclophosphamide, cyclosporine, and high-dose intravenous immunoglobulins [1]. The standard first-line approach, preferred by most dermatologists, involves systemic corticosteroids (prednisolone, 1.0–1.5 mg/kg/day) combined with corticosteroid-sparing immunosuppressive agents, mainly azathioprine and mycophenolate mofetil [1].

A multitude of vaccines, such as messenger ribonucleic acid (mRNA) vaccines (Pfizer/BioNTech (BNT162b2) and Moderna (mRNA-1273)), adenovirus viral vector vaccines (Oxford-AstraZeneca (AZD1222)), the whole inactivated virus vaccine from Sinovac (CoronaVac), and others have been developed to counteract the severe acute respiratory syndrome coronavirus 2 (SARS-CoV-2) infection [2,7]. The vaccination drive against the coronavirus disease 2019 (COVID-19) pandemic stood as a pivotal endeavor for healthcare systems, characterized by an exceptionally favorable risk–benefit ratio [7,8]. Nonetheless, a range of vaccine-related adverse reactions has been documented in randomized controlled trials, mostly of mild to moderate severity. These include headache, muscle pain, joint discomfort, fatigue, chills, fever, and localized redness or swelling at the injection site [7,9]. In contrast, severe adverse events, such as allergic reactions or anaphylaxis, are infrequent, occurring at rates between 0.2% and 0.3% [7,9,10]. Additionally, SARS-CoV-2 vaccination has been linked to various dermatological reactions, from localized issues, like swelling, erythema, and delayed hypersensitivity, to broader manifestations, such as urticaria, erythema multiforme, and vasculitis [7,9,11]. Recent studies suggest that SARS-CoV-2 vaccines may potentially trigger or exacerbate AIBDs [7,9,10,11,12,13,14]. The existing literature describes cases of the new onset or relapse of pemphigus vulgaris (PV) and pemphigus foliaceus (PF) following COVID-19 vaccination; however, the actual number of affected individuals might be higher due to incomplete data collection [3,7,15,16,17,18].

The pathophysiological mechanisms are likely attributed to the resemblance between the RNA antigen present in these vaccines and the cellular nuclear matter [14,19,20,21,22,23]. The complex immunological interplay between the COVID-19 vaccine and AIBDs is highlighted by autoimmunity: vaccines could trigger autoimmunity, whereas AIBDs are the result of dysregulated autoimmunity. Induced autoimmunity is believed to be the underlying mechanism for the new onset or exacerbation of dermatologic disorders following SARS-CoV-2 infection [14,19,20,21,22,23]. This process involves molecular mimicry: the newly generated antiviral antibodies also target self-antigens, potentially leading to immune-mediated dermatologic diseases [14,19,20,21,22,23]. Additionally, the immune dysregulation caused by SARS-CoV-2 infection can result in hypersensitivity reactions, which contribute to the development of cutaneous lesions [14,19,20,21,22,23].

Our review seeks to offer a comprehensive overview of the reported cases of PF developed after COVID-19 vaccination, delving into their intricate presentation. This investigation is focused on enhancing the comprehension of the pathophysiological mechanisms underlying the condition, emphasizing the critical need for vigilant monitoring of PF following mRNA vaccination.

## 2. Existing Data on PF Onset after SARS-CoV-2 Vaccination

We conducted a literature review of new-onset PF following anti-SARS-CoV-2 vaccination, detailing the demographic, clinical, and immunological characteristics of the presented patients. Notably, PF is less prevalent than PV. Thirteen PF case reports have been documented globally [24,25,26,27,28,29,30,31,32] (Table 1). These case reports, spanning multiple nations and encompassing diverse underlying diseases and immunization regimens, describe previously healthy individuals developing new-onset PF.

Prior to COVID-19, sporadic PF was considered a rare occurrence, accounting for 20–30% of all pemphigus cases. The estimated annual incidence of sporadic PF in the USA and Europe is less than 1 case per million inhabitants [33,34]. Genetic studies have identified an association between sporadic PF and specific human leukocyte antigen (HLA) alleles in different populations [33]. Among Mexican patients, an association with HLA-DRB10101 was observed [33,35]. In other populations, such as Brazilian, Dutch, French, and Italian, HLA-DRB104 was linked to an increased risk of developing PF [33,36,37,38].

The majority of PF cases worldwide occurred in individuals aged 50 to 83 years [24,25,26,27,28,29]. The median age of patients in the PF cohort is 60 years (interquartile range, 50–83 years), with no notable gender predilection (six females, seven males) [7,24,27]. This slight deviation from the typical age range for PF patients, as reported prior to and after the COVID-19 pandemic, points to potential contributing factors [24]. Notably, polydrug usage is prevalent among older adults, complicating the identification of the primary cause of PF [24]. As a result, the age difference is related to the COVID-19 vaccine as opposed to the prior case reports that described medication-related cases.

There is accumulating evidence linking new-onset or worsening AIBDs following SARS-CoV-2 vaccination [39]. A systematic meta-analysis conducted by Hinterseher et al. on six autoimmune and inflammatory skin diseases supports this association [39]. To mitigate the bias of unrelated diagnoses or disease exacerbations, the meta-analysis focused only on individual cases occurring within 21 days post-vaccination [39]. No significant difference was observed between vector-based and mRNA-based vaccines in relation to the new onset or exacerbation of AIBDs [39]. However, mRNA-based vaccines exhibited a higher incidence of de novo or relapsing AIBDs, likely due to their more frequent use compared to other vaccine types [39]. Indeed, most cases reported in the literature share a common vaccination regimen involving the BNT162b2 COVID-19 vaccine [7,24]. According to Calabria et al., among all AIBD cases, the majority of PF cases (22 [62.9%]) emerged after administration of the BNT162b2 vaccine, six cases (17.1%) post-mRNA-1273 administration, three (8.6%) after AZD1222 administration, and another three cases (8.6%) following CoronaVac administration [7,27]. Calabria et al.’s investigation is consistent with previous studies suggesting that the BNT162b2 vaccine has a higher association rate with AIBD onset, potentially due to its extensive utilization in the Western world population compared to other vaccines [7,27]. Therefore, the actual incidence of new-onset or worsening AIBDs post-SARS-CoV-2 vaccination may be underestimated [39].

A further distinguishing characteristic is the number of vaccine doses administered before the onset of PF. Data from the literature suggest looking for pemphigus symptoms between 3 days and 3 weeks after receiving a COVID-19 vaccine, whether the initial or subsequent dose [3]. Among the reviewed case reports, AIBDs developed 2 days to 2 months after the administered dose, consistent with observations from earlier case studies [24]. Notably, all previously reported cases were de novo PF. Most of the reported cases appeared after the second dose of the BNT162b2 vaccine. No reactivation cases were reported among the analyzed cases.

Pham et al. documented two case reports of PF occurring after COVID-19 vaccination and utilized the Najaro scale to evaluate the likelihood that the vaccines contributed to the onset of the disease [24,26,40]. The vaccines were identified as probable contributors to PF in these cases, with Najaro scores of 3 and 7, respectively [24,40]. Currently, there is no definitive method for confirming the role of medications in drug-induced reactions beyond questionnaire-based assessments, making it challenging to identify the specific drug responsible for the adverse effects reported in these cases [24]. Although the precise etiology remains unclear, aberrant immune responses are implicated in pemphigus development, with any stimulation of the immune system posing a potential risk [18].

Notably, all documented cases of COVID-19 vaccine-induced PF worldwide exhibited clinical features consistent with typical PF manifestations, including transient, flaccid bullae, accompanied by pain and pastry-like exfoliation evolving into crusty erosions on an erythematous base, primarily on the seborrheic areas. The differential expression patterns of desmoglein isoforms in various tissues and the profile of anti-desmoglein IgG explain the blisters’ localization [24]. The diagnosis and confirmation of AIBDs entail comprehensive clinical, histological, and immunochemical evaluations.

Cojocaru et al. reported the case of a 34-year-old Caucasian female who developed PF during the third trimester of pregnancy, 5 days after receiving the second dose of the BNT162b2 COVID-19 vaccine [2]. The new onset of PF in the postpartum period is exceedingly rare, but a couple of cases have been previously reported in the literature [41]. Thus, it may be difficult to differentiate between PF triggered by COVID-19 vaccination and PF triggered by pregnancy. Several clinical, immunological, and temporal factors should, therefore, be taken into consideration [41,42,43]. Both the patient’s past medical history and family history of any previous autoimmune conditions or recent vaccinations may increase the likelihood of an autoimmunity diagnosis [41,42,43]. Assessing the timing of PF symptoms can be also useful: PF triggered by COVID-19 typically follows a recent infection or immunization, while pregnancy-related PF generally occurs during the first or second trimester of pregnancy or shortly after pregnancy [41,42,43]. A strict follow-up of serological markers and clinical manifestations in genetically predisposed patients may be beneficial to differentiate between PF triggered by COVID-19 infection or vaccination and PF triggered by pregnancy [41,42,43].

The majority of patients (85%) diagnosed with COVID-19 vaccine-induced PF experienced rapid improvement and overall good clinical response following short-term therapy, typically after 2–4 weeks. Corticosteroids (oral and topical) were commonly employed in conjunction with other immunosuppressive agents, such as azathioprine, mycophenolate mofetil, and rituximab [24]. PF was a rare vaccination-associated side effect, and it was effectively controlled with immunosuppressive therapies.

However, it is essential to acknowledge that PF cases definitively attributed to vaccines sometimes have insufficient evidence to make a certain association with vaccination.

## 3. Discussion

In response to the global pandemic, COVID-19 vaccines have emerged as essential tools in preventing infection and conferring protective immunity [3]. Generally, they have been deemed both safe and well-tolerated, although side effects have been reported [3].

The potential link between vaccination and AIBDs has been previously discussed in research papers [44,45]. There is still a current need for high-quality, extensive research studies to prove a strong association between these two entities. The onset or reactivation of pemphigus may be causally related to vaccination, induced by immunosuppressive medications, or connected to the pathogenic relationship between viral infection and immune dysregulation, resulting in autoimmunity [3,46,47,48]. Although the precise etiology remains unclear, aberrant immune responses are implicated in pemphigus development, with any immune system stimulation posing a potential risk [18].

The literature shows that both vaccinations with vaccines containing viral genetic material and viral infections can trigger autoimmune reactions in predisposed individuals [24,49]. Virus- and vaccine-associated autoimmunity is a well-established phenomenon in immunology [50,51]. Numerous viruses and vaccines have been documented to induce autoimmune responses, often due to cross-reactivity between viral or vaccine antigens and host tissues or as a result of adjuvant effects [7,33].

While the temporal relationship between vaccination and pemphigus onset in our case might appear coincidental, the tight chronological sequence raises suspicions [7,18]. In some case series, the timing of pemphigus onset is the sole factor suggesting a causal relationship [7,18,27]. Correlation between these events should be corroborated by larger population-based studies examining variations in pemphigus incidence and other AIBDs before and after widespread vaccine administration [7,27]. Although the causality between SARS-CoV-2 vaccination and new-onset PF reported in some cases remains uncertain, several factors, including the lack of prior cutaneous disease, symptom onset within 1 month of vaccination, the propensity of vaccines to trigger immune-mediated skin conditions, and the rarity of pemphigus, suggest that the observed relationship may not be just coincidental [7,31,52]. Despite the observed temporal correlation, it is important to note that a definitive cause–effect relationship has not been established.

### 3.1. The Immunological Interplay between COVID-19 Vaccines and AIBDs

mRNA vaccines represent a groundbreaking approach to vaccination, with SARS-CoV-2 being the inaugural pathogen targeted by this technology on a wide scale [53,54]. Both available COVID-19 vaccines of this class harness mRNA encoding the SARS-CoV-2 spike protein, a crucial component responsible for viral binding to the host receptor angiotensin-converting enzyme 2 and subsequent cellular internalization [53]. This unique vaccine strategy capitalizes on the body’s innate immune pathways to orchestrate a response akin to that provoked by natural viral infections [32,53,54,55,56]. 

Following the onset of the global COVID-19 vaccination campaign, there have been increased reports of de novo autoimmune diseases emerging post-vaccination. SARS-CoV-2 infection has been linked to autoantibody development, prompting questions about whether COVID-19 vaccines might also induce autoantibodies, especially in individuals with autoimmune conditions [57]. The origin of these antibodies is not yet fully understood [57]. Nevertheless, potential mechanisms inherent to the SARS-CoV-2 spike protein suggest that vaccines targeting this antigen may also induce humoral autoimmunity [57].

In addition to molecular homology and cross-reactivity, HLA allelic variants are pivotal in the onset of autoimmunity [22]. Talotta et al.’s computational analysis suggests that certain epitopes from the BNT-162b2 mRNA vaccine can be effectively presented by nucleated cells and antigen-presenting cells (APCs) due to preferential binding to specific class I or class II HLA molecules [22]. The research indicates that HLA polymorphisms may also affect the clinical progression of COVID-19 or the response to the vaccine by modulating epitope presentation to protective T cells [22].

Consequently, the BNT-162b2 mRNA vaccine’s protein product includes immunogenic epitopes that could induce autoimmune phenomena in susceptible individuals through various mechanisms, such as the activation of pattern recognition receptors (PRRs), molecular mimicry, the polyclonal stimulation of B cells, autoinflammation, and type I interferon production [22,44,45]. Genotyping for HLA alleles could aid in identifying individuals at risk [22]. However, further studies are needed to elucidate the immunopathogenic role of COVID-19 vaccines in the context of autoimmune diseases.

AIBDs arise from dysregulated immune responses targeting skin structures [24]. IgG autoantibodies targeting desmoglein-1 and -3, the pivotal desmosome adhesion molecules, are hallmark features characterizing the spectrum of pemphigus autoimmunity [24]. The involvement of T and B lymphocytes in the pathogenesis of these diseases underscores their immunological complexity [17,24].

One proposed theory suggests that molecular mimicry between specific basement membrane proteins and the SARS-CoV-2 spike protein may trigger autoimmune blistering diseases (AIBDs) [44,58]. Furthermore, mRNA vaccines might activate pro-inflammatory pathways through interactions with Toll-like receptors [44]. This interaction could result in increased cytokine production, including interleukin (IL)-4, IL-17, interferon-γ, and tumor necrosis factor-α [44,59,60,61]. Given that autoreactive T cells and imbalances in T helper (Th)1 and Th2 responses play a significant role in the pathogenesis of pemphigus, the cytokine changes induced by the vaccine could lead to an imbalance in Th2 responses against skin antigens [44,62]. This imbalance may facilitate the development of autoreactive B cells and contribute to AIBDs [44,61]. Additionally, inflammation caused by the vaccine may damage the basement membrane, resulting in the formation of anti-basement membrane antibodies [44,58]. 

HLA molecules, such as alleles HLA-DQB10503 and HLA-DRB10402 associated with pemphigus, might also be significant factors in drug-induced AIBDs [44,63]. 

Nevertheless, the current research has not definitively established these mechanisms for vaccine-induced autoimmune disorders [3]. Furthermore, Martino et al. contend that vaccines are not a causative factor for autoimmune diseases [64]. The authors state that in many instances, the presence of autoantibodies is temporary and does not result in any clinical outcomes [64]. The typically brief and self-limiting nature of such autoimmune responses suggests a generally favorable prognosis, indicating that these immunological reactions are transient [64]. Furthermore, they conclude that the occurrence of autoimmunity does not automatically lead to the development of an autoimmune disease [64]. The immune system’s robust regulatory mechanisms usually prevent these autoimmune responses from progressing into full-blown autoimmune disorders. Therefore, the review highlights that while there are occasional reports of autoimmune conditions following vaccination, the evidence linking vaccines directly to autoimmune diseases is generally weak and does not establish a clear causal relationship [64]. The authors argue that these cases are often anecdotal and do not establish a clear causal relationship. They highlight the need for robust, large-scale studies to further investigate any potential links [64].

### 3.2. Mechanisms of Vaccine-Induced Skin Findings

Various mechanisms of vaccine-induced skin findings have been thoroughly discussed, including vaccine-spike glycoprotein-induced reactions, delayed hypersensitivity response to vaccination, T cell-mediated response resulting from molecular mimicry to viral epitopes, off-target immune activation post-vaccination (that could explain bullous pemphigoid and leukocytoclastic vasculitis) and RNA-mediated activation of innate immunity via Toll-like receptors that could also increase type I interferon release and explain pernio/chilblains presentations.

The precise mechanisms underlying the development of autoimmune diseases following antiviral vaccination are still a subject of debate [3]. 

Molecular mimicry, along with subsequent immunological cascades targeting nuclear components in predisposed individuals, is frequently proposed as a mechanism for triggering autoimmunity in these conditions. Additionally, inflammatory dysregulation and the bystander effect are also considered significant contributors to the development of these autoimmune disorders [30,63]. Consequently, the pathogenesis of AIBDs following COVID-19 immunization is anticipated to follow a similar immunological pattern [30].

#### 3.2.1. T Cell-Mediated Response Resulting from Molecular Mimicry to Viral Epitopes

One proposed mechanism is immune cross-reactivity to vaccine antigens, including adjuvants, induced by molecular mimicry and epitope spread, particularly in genetically predisposed individuals [3,24,31,32,39,65]. 

Vaccine-induced components can act as foreign antigens, prompting the production of cross-reactive antibodies against both the foreign antigen and desmoglein-1 [18,66]. Molecular mimicry refers to the cross-reactivity between a foreign antigen and a self-antigen, leading to a breakdown of self-tolerance and the onset of autoimmunity [24,32,39,65]. Additionally, epitope spreading occurs when the immune response against self-antigens is activated by the release of these antigens during ongoing autoimmune reactions [32]. Neutralizing antibodies against SARS-CoV-2 have been observed in 18% of individuals on the seventh day post-vaccination, potentially elucidating the onset of pemphigus around this time [18,67]. This phenomenon has been proposed to account for various instances of pemphigus triggered by bacterial, viral antigens, or pharmacological agents [33].

The BNT-162b2 mRNA vaccine has been associated with the onset of autoimmunity in susceptible individuals due to the presence of certain protein epitopes [22]. Talotta et al. conducted an in-silico analysis, identifying a total of 5693 epitopes related to 21 viral and human proteins [22]. These human proteins included CHL1, ENTPD1, SLC35G2, MEAF6, and ZFHX2 [22]. 

Importantly, some autoepitopes can be presented by HLA alleles, potentially mimicking self-epitopes or binding to HLA alleles linked to a higher risk of specific autoimmune diseases in certain ethnic groups [22]. These data suggest that the post-vaccination autoimmune phenomena documented in the literature may be due to cross-reactive antibodies or T cells targeting self-epitopes sharing similarities with the epitopes from the BNT-162b2 vaccine spike protein.

Such epitopes can be recognized by T-cell receptors (TCRs), triggering the activation of effector T cells [22]. Polymorphic HLA genes are known to encode isoforms of the major histocompatibility complex (MHC) molecules with amino acid substitutions at key positions, such as peptide binding sites [22,68]. These variations can significantly influence thymic selection centrally or affect the binding affinity of epitopes peripherally, thereby expanding the range of recognized allo- and autoepitopes and enlarging the pool of reactive T and B cells [22,68,69]. Consequently, HLA polymorphisms may enhance immune defenses against pathogens but also increase the risk of autoimmune diseases [22,68,69]. Additionally, HLA genes can influence the survival of T cells with specific patterns in the complementarity determining region 3 (CDR3) of the TCR, which are characterized by a higher ability to present autoepitopes [22,68,69].

Even if desmogleins were not specifically listed among the identified proteins in the Talotta et al. study, some of the other proteins found might have structural or sequence similarities to desmogleins [22]. This similarity can lead to molecular mimicry, where vaccine epitopes resemble self-epitopes, potentially triggering an autoimmune response [22]. The presence of numerous epitopes suggests a broad immune response that might include cross-reactivity with proteins not directly identified in the study. Additionally, the vaccine-induced autoimmune response might involve indirect mechanisms, such as the activation of autoreactive T or B cells, which could then target desmogleins or other basement membrane components due to the initial immune activation caused by the vaccine. Further research is needed to clarify these mechanisms and validate the role of molecular mimicry in vaccine-induced autoimmune conditions like PF.

However, a recent case series by Kasperkiewicz et al., which included 24 healthy individuals with prior SARS-CoV-2 infections or vaccinations but no history of autoimmune bullous disorders, found no evidence of cross-reactivity between anti-SARS-CoV-2 antibodies and pemphigus autoantigens in serum [39,70]. The study’s limitations, including its small sample size and likely lack of genetically predisposed participants, suggest that molecular mimicry alone may not fully account for the development of pemphigus following SARS-CoV-2 vaccination [19,27,31].

#### 3.2.2. Bystander Activation of Immune Cells

Other pathophysiologic mechanisms, such as the nonspecific bystander activation of immune cells or epitope mapping studies, have been investigated [3,31].

Bystander activation occurs when self-epitopes from damaged host tissue stimulate the proliferation of antigen-presenting cells and subsequently, autoreactive T cells [3,31]. After bystander activation, epitope spreading ensues, wherein the immune system reacts to different parts of the same or a different protein, thereby diminishing the likelihood of the pathogen evading immune detection with a single mutation in an immunogenic epitope [3,31]. These immune responses, triggered by either natural infection or artificial antigens from vaccines, can potentially compromise the immune system, ultimately leading to an autoimmune disorder [3]. Hence, it is conceivable to speculate that the COVID-19 vaccine might trigger pemphigus, but only if autoreactive T and B cells are activated and self-epitopes are exposed [3].

Recent case reports have highlighted potential associations between the BNT162b2 vaccine and various immune-mediated dermatologic afflictions, such as vitiligo and erythematous rashes. These conditions may be related to the stimulation of Toll-like receptors by mRNA vaccines [3,31]. 

#### 3.2.3. RNA-Mediated Activation of Innate Immunity

Moreover, a hyperimmune reaction could foster autoantibody formation against the 160-kd antigen (desmoglein-1) likely occurring on a genetic predisposition background [6,18].

Some researchers have proposed a potential link between autoimmune blistering diseases (AIBDs) and COVID-19 vaccination, suggesting that transient immune activation could occur in individuals who are already predisposed to subclinical autoimmunity [15,27]. This hypothesis posits that a broad, nonspecific immune mechanism might account for the range of immune-mediated conditions observed post-vaccination, from autoinflammatory disorders to autoimmune manifestations, which can affect multiple systems or be specific to certain organs, such as pemphigus [15,27]. Additionally, the frequency of immune-mediated diseases seems higher in those with pre-existing rheumatic or autoimmune conditions, highlighting the role of individual susceptibility [27,71]. 

Both the BNT162b2 and ChAdOx1 vaccines initiate innate inflammatory responses through distinct mechanisms: the mRNA in BNT162b2 acts as an adjuvant, binding to Toll-like receptors and enhancing type I interferon (IFN) production, along with activating RNA sensors, such as Toll-like receptor 7, and components of the inflammasome-like melanoma differentiation-associated protein 5 (MDA5) and nucleotide-binding oligomerization domain-containing protein 2 (NOD2) [33,36,71,72]. Type I IFN is known for its role in promoting antigen presentation, B-cell differentiation, and IgG secretion [33,73,74,75,76].

The Moderna mRNA-1273 vaccine may additionally stimulate the production of interferon γ, interleukin (IL)-17, and tumor necrosis factor α cytokines, potentially contributing to the development of pemphigus through T cell-dependent pathways [60,77]. Conversely, the mRNA BNT162b2 vaccine is thought to indirectly promote the production of IL-4, IL-17, and IL-21 cytokines, which have associations with autoimmune disorders [59,77].

The pathogenesis of pemphigus involves a complex interaction among TH2, TH17, and TFH17 cells, with significant roles played by IL-4, IL-17, and IL-21 [3,78]. Although IL-4 production has been noted following COVID-19 vaccination, there is a lack of data regarding the levels of IL-17 and IL-21 in individuals who develop pemphigus post-vaccination [3].

Sahin et al. conducted a clinical trial involving the BNT162b1 vaccine, which utilizes mRNA combined with lipid nanoparticles and modified nucleosides [3,76]. This vaccine encodes the receptor-binding domain (RBD) of the SARS-CoV-2 spike protein [3,76]. The study demonstrates that BNT162b1 elicited a strong T-cell response, marked by an expansion of RBD-specific CD4+ and CD8+ T cells [3,76]. It also led to the production of high levels of inflammatory cytokines, such as IFN-γ and IL-2, resulting in a T helper type 1 (TH1)-biased T-cell response [3,76]. In the majority of the 52 participants, RBD-specific CD4+ and CD8+ T cells were observed to produce IFN-γ, IL-2, or both [3,76]. Importantly, IL-4 production was generally absent, with only one participant exhibiting notable IL-4 secretion following BNT162b1 vaccination [3,76]. This suggests that the pathogenic role of TH2 responses in vaccine-induced pemphigus may be unlikely [3,76].

The activation of Toll-like receptors on antigen-presenting cells has been proposed as a potential mechanism for the development of autoimmune diseases following vaccination [39,79]. The double-stranded DNA in the AZD1222 adenovirus vector vaccine is believed to activate Toll-like receptor 9, leading to elevated levels of type I interferons that could potentially induce pemphigus-related autoimmunity [55,77]. However, some reports challenge this view, indicating that vaccine developers have incorporated stabilizing adjuvants and removed interferon-inducing double-stranded messenger RNA from the final formulations to reduce the risk of triggering autoimmune reactions [55,77].

## 4. Future Perspectives on mRNA Vaccines 

mRNA vaccines, which have been developed and tested in preclinical models against various pathogens, are stirring significant interest due to their innovative approach and potential benefits [80]. Unlike traditional vaccines that use recombinant proteins or whole viral particles, mRNA vaccines come with a host of advantages. For one, the single-stranded RNA (ssRNA) acts as its own adjuvant, negating the need for additional adjuvants [80,81]. This allows the vaccine to leverage the body’s cellular machinery to produce the antigen in a way that mimics natural viral infection, ensuring proper folding and surface presentation of the antigen [81]. Additionally, mRNA vaccines offer a safer profile compared to DNA-based vaccines because they present a lower risk of host genome integration [80].

The adaptability of mRNA technology makes it a game-changer for a wide array of infectious diseases [80]. Unlike conventional vaccines that require specific biosafety conditions for pathogen cultivation and inactivation, mRNA vaccines bypass these requirements, making them easier to produce and adapt [80,82]. This flexibility means that the same manufacturing process can be streamlined and repurposed to address various global health threats swiftly [80].

In the realm of cancer treatment, mRNA vaccines are also showing remarkable potential. By encoding tumor-specific antigens, these vaccines could prime the immune system to recognize and attack cancer cells more effectively [73,83]. In preclinical studies involving murine models, mRNA vaccines have demonstrated their ability to activate robust immune responses against tumors [73,83]. Clinical trials are advancing our understanding of these vaccines’ efficacy, with studies like KEYNOTE-942 exploring the integration of mRNA-4157/V940 into melanoma treatment protocols. This includes examining its use alongside immune checkpoint inhibitors to enhance therapeutic outcomes [73,83].

Recent advancements in our understanding of the influence of untranslated mRNA sequences, formulations, and injection technologies suggest promising developments in mRNA vaccine technology in the coming years [80]. This innovative technology has opened up novel possibilities for developing vaccines against influenza, zika, and the human immunodeficiency virus. Furthermore, emerging technologies, such as machine learning and artificial intelligence, are expected to provide new insights into mRNA vaccine design, leading to the development of improved product candidates [80].

Therefore, several strategies can be taken into consideration to further prevent an autoimmune disease in patients, such as performing thorough pre-vaccination screenings to identify individuals with a history of autoimmune affliction or genetic predisposition, including their family medical history and potentially HLA typing to identify known susceptibility alleles; the assessment of the risk–benefit ratio of vaccination for individuals with pre-existing autoimmune conditions and the customization of vaccine recommendations according to their individual risk profiles; the use of adjuvants that have a lower likelihood of triggering autoimmune responses, focusing on researching and developing new vaccines that boost immune responses without causing excessive immune system stimulation; the establishment of comprehensive post-vaccination monitoring systems to identify early signs of autoimmune reactions; the encouragement of healthcare providers and patients to promptly report any adverse events; the development of personalized vaccination schedules, schemes, and dosages based on individual immune profiles and medical histories; the education of both healthcare professionals and patients about the potential risks of vaccine-induced autoimmunity and the importance of reporting unusual symptoms following vaccination; the development of anti-inflammatory strategies to mitigate inflammation caused by vaccines (the co-administration of anti-inflammatory agents in high-risk individuals); and the provision of diligent follow-up care for patients after vaccination, particularly for those with a known predisposition to autoimmune disorders and early intervention to reduce the severity of autoimmune responses [84,85,86,87,88].

## 5. Conclusions

Our findings suggest a potential association between new-onset PF and COVID-19 vaccination, which may enhance or trigger the immunological response, as seen in other AIBDs. According to our knowledge, the association of PF is less prevalent when compared with other AIBDs induced by COVID-19 vaccination. 

The complex immunological interplay between the COVID-19 vaccine and AIBDs is highlighted by autoimmunity: vaccines cause autoimmunity, whereas AIBDs are the result of dysregulated autoimmunity. Virus- or vaccine-associated autoimmunity is well-documented, with viruses and vaccines proposed to trigger various autoimmune responses. Susceptible individuals with a predisposition to autoimmune/autoinflammatory dysregulation may be at higher risk of immunological side effects after nucleic acid-containing vaccines. However, establishing a cause–effect relationship is challenging, though the presence of a temporal correlation may suggest an association.

Raising awareness about potential adverse effects linked to SARS-CoV-2 vaccination is essential, particularly as new cases continue to emerge. It is essential for clinicians to remain vigilant about the possible side effects, including the potential onset or exacerbation of dermatological conditions following vaccination. Prompt recognition and treatment of these conditions are key. However, the latency period and the identification of the specific vaccine responsible can pose significant challenges. While vaccination remains a critical tool in combating COVID-19, it is equally important to educate patients about monitoring and reporting any skin changes that occur after receiving the vaccine. This approach ensures that any adverse effects can be addressed promptly without discouraging vaccination efforts.

## Figures and Tables

**Table 1:** The reported cases of PF following COVID-19 vaccination, including the cases described in our study.

Authors	Country	Number of Cases	Age	Sex	Vaccine Regimen	Onset Milestones	Concomitant Drugs	Histopathology (DIF)	Treatment	Clinical Response
Cojocaru et al. [2]	Romania	1	34	F	2 doses of BNT162b2	5 days after 2nd dose	None	Acantholysis at the granular layer (epidermal reticular deposition of IgG and C3, with the absence of IgM, IgA, and fibrinogen	Oral corticosteroid	Complete response after 4 weeks
Pham et al. [24]	Vietnam	2	53	F	Mixed-3 doses of BBIBP-CorV followed by 1 dose of AZD1222	3 weeks after 4th AZD1222 dose in the mixed regimen	Amlodipine	Acantholysis above the stratum basalis, dermal lymphocyte, and neutrophil infiltration (intraepidermal IgG and C3)	Corticosteroid and rituximab	Almost complete response in 3 weeks
			30	F	2 doses of mRNA-1273	2 months after 2nd mRNA-1273 dose	None	Acantholysis above the stratum basalis (intraepidermal IgG and C3)	Topical and systemic corticosteroid	Almost complete response in 4 weeks
Corrá et al. [27]	Italy	2	80	M	3 doses of BNT162b2	17 days after 3rd dose	Amiloride, hydrochlorothiazide, esomeprazole	Subcorneal acantholysis with neutrophilic infiltration within the blister (PT1: negative; PT2: intercellular IgG deposits)	Oral corticosteroid, rituximab, mycophenolate	Probably good clinical response
			66	F	2 doses of BNT162b2	4 weeks after 2nd dose	Rabeprazole, ticlopidine, atorvastatin, amlodipine, hydrochlorothiazide	Subcorneal acantholysis with neutrophilic infiltration within the blister (PT1: negative; PT2: intercellular IgG deposits)	Oral corticosteroid, rituximab, mycophenolate	Probably good clinical response
Pourani et al. [25]	Iran	1	75	M	3 doses of BBIBP-CorV	2 weeks after 3rd dose	None	Superficial epidermal bullae, mild spongiosis, superficial dermal perivascular inflammation (intraepidermal IgG and C3)	Topical corticosteroid, rituximab	Significant response in 4 weeks
Lua et al. [26]	Singapore	1	83	M	2 doses of BNT162b2	2 days after 2nd dose	N/A	Subacute spongiotic dermatitis (C3 dermal-epidermal junction and intercellular deposition)	Prednisolone	Good clinical response
Hali et al. [28]	Morocco	1	50	F	2 doses of BNT162b2	15 days after 2nd dose	None	Superficial epidermal blistering process, intact basal layer, intraepidermal eosinophils (intracellular IgG and C3)	Oral corticosteroid	Complete response in 3 weeks
Yıldırıcı et al. [29]	Turkey	1	65	M	2 doses of BNT162b2 (6 weeks apart)	1 month after 1st dose; 2 weeks after 2nd dose	Nebivolol, valsartan-hydrochlorothiazide	Intraepidermal acantholytic blister, abundant neutrophils, and scarce eosinophils (intercellular IgG and C3)	Oral corticosteroid, azathioprine	Marked response in 2 weeks
Rouatbi et al. [30]	Tunisia	2	70	M	Mixed-2 doses of CoronaVac followed by 1 dose of BNT162b2	7 days after BNT162b2 dose	N/A	Intraepidermal acantholytic blister (intercellular IgG and C3 within the epidermis)	Prednisone, Clobetasol	Good clinical response in 3 weeks
			48	M	1 dose of AZD1222	5 days after 1st dose	None	Superficiel cleft within the epidermis with acantholysis (intercellular IgG and C3 within the epidermis)	Prednisone, Clobetasol	Good clinical response in 2 weeks
Gui et al. [31]	California	1	67	F	2 doses of mRNA-1273	2 weeks after 2nd dose	N/A	Intraepidermal acantholysis (intercellular IgG and C3)	Prednisone, Clobetasol and Mupirocin	Almost complete response in 8 weeks
Alami et al. [18]	Morocco	1	44	M	2 doses of BBIBP-CorV	7 days after 1st dose	None	Acantholysis with superficial intra-epidermal cleavage (intercellular IgG)	Prednisone, Azathioprine	No good clinical response
Reis et al. [32]	Portugal	1	35	F	2 doses of BNT162b2 (6 weeks apart)	2 weeks after 2nd dose	None	Acantholitic keratinocytes and detachment of the stratum corneum (intercellular deposition of IgG and C3)	Prednisolone, Clobetasol	Good clinical response in 4 weeks

## Data Availability

This review summarizes data reported in the literature, and it does not report primary data.
